# Impaired behavioural pain responses in *hph-1* mice with inherited deficiency in GTP cyclohydrolase 1 in models of inflammatory pain

**DOI:** 10.1186/1744-8069-9-5

**Published:** 2013-02-19

**Authors:** Arafat Nasser, Ole J Bjerrum, Anne-Marie Heegaard, Anette T Møller, Majbritt Larsen, Louise S Dalbøge, Erik Dupont, Troels S Jensen, Lisbeth B Møller

**Affiliations:** 1 Department of Drug Design and Pharmacology, Faculty of Health and Medical Sciences, Copenhagen University, Copenhagen, Denmark; 2 The Danish Pain Research Center, Århus University Hospital, Århus, Denmark; 3 Applied Human Molecular Genetics, Kennedy Center, Copenhagen University Hospital, Rigshospitalet, Glostrup, Denmark; 4 Department of Neurology, Århus University Hospital, Århus, Denmark

**Keywords:** GTP-cyclohydrolase, Tetrahydrobiopterin, *hph-1*, Inflammatory pain, Mechanical hypersensitivity, CFA, Formalin, Capsaicin, Dystonia

## Abstract

**Background:**

GTP cyclohydrolase 1 (GTP-CH1), the rate-limiting enzyme in the synthesis of tetrahydrobiopterin (BH4), encoded by the *GCH1* gene, has been implicated in the development and maintenance of inflammatory pain in rats. In humans, homozygous carriers of a “pain-protective” (PP) haplotype of the *GCH1* gene have been identified exhibiting lower pain sensitivity, but only following pain sensitisation. *Ex vivo*, the PP *GCH1* haplotype is associated with decreased induction of *GCH1* after stimulation, whereas the baseline BH4 production is not affected. Contrary, loss of function mutations in the *GCH1* gene results in decreased basal *GCH1* expression, and is associated with DOPA-responsive dystonia (DRD). So far it is unknown if such mutations affect acute and inflammatory pain.

**Results:**

In the current study, we examined the involvement of the *GCH1* gene in pain models using the hyperphenylalaninemia 1 (*hph-1*) mouse, a genetic model for DRD, with only 10% basal GTP-CH1 activity compared to wild type mice. The study included assays for determination of acute nociception as well as models for pain after sensitisation. Pain behavioural analysis of the *hph-1* mice showed reduced pain-like responses following intraplantar injection of CFA, formalin and capsaicin; whereas decreased basal level of GTP-CH1 activity had no influence in naïve *hph-1* mice on acute mechanical and heat pain thresholds. Moreover, the *hph-1* mice showed no signs of motor impairment or dystonia-like symptoms.

**Conclusions:**

In this study, we demonstrate novel evidence that genetic mutations in the *GCH1* gene modulate pain-like hypersensitivity. Together, the present data suggest that BH4 is not important for basal heat and mechanical pain, but they support the hypothesis that BH4 plays a role in inflammation-induced hypersensitivity. Our studies suggest that the BH4 pathway could be a therapeutic target for the treatment of inflammatory pain conditions. Moreover, the *hph-1* mice provide a valid model to study the consequence of congenital deficiency of *GCH1* in painful conditions.

## Background

Chronic pain is a severe and common healthcare problem that affects millions of people worldwide. In spite of a number of analgesics available for pain therapy, pain often remains inadequately treated in many patients and represents a major cause of suffering and reduced quality of life [1]. One of the reasons for the difference in success of pharmacologic pain treatment rely on the different genetic disposition of patients to develop pain or to respond to analgesics [2].

The *GCH1* gene is one of few genes reported to be involved in the modulation of pain sensitivity in humans [3-5]. The gene codes for the enzyme guanosine triphosphate cyclohydrolase 1 (GTP-CH1), the first and rate-limiting enzyme in the *de novo* synthesis of 5,6,7,8-tetrahydrobiopterin (BH4) (see Additional file 1). BH4 is an essential cofactor for phenylalanine, tyrosine and tryptophan hydroxylases and for all isoforms of nitric oxide synthase (NOS). Hence, BH4 regulates the synthesis of catecholamines, serotonin and nitric oxide (NO) (see Additional file 1), all involved in pain signalling [6]. In 2002, Costigan and colleagues made a systematic search for pain-related genes using microarray based gene expression analysis [7]. This led to the discovery of two genes both involved in BH4 biosynthesis; the *GCH1* gene and the gene for sepiapterin reductase (*SR*) (see Additional file 1). Both genes were upregulated in dorsal root ganglion (DRG) following peripheral axotomy in rats. In a follow-up study, BH4 synthesis was found to be upregulated in primary sensory neurons after peripheral inflammation and nerve injury [4]. Inhibition of BH4 synthesis with the prototypic GTP-CH1 inhibitor 2,4-diamino-6-hydroxypyrimidine (DAHP) reduced the nociceptive responses in rodent models of neuropathic and inflammatory pain accompanied by reduced BH4 concentrations in DRGs, whereas intrathecal injection of BH4 induced pain in naïve rats [4]. Furthermore, in humans a particular haplotype of the *GCH1* gene identified as “pain protective”(PP) was found to be associated with reduced pain sensitivity in healthy subjects as well as persistent low back pain in patients after discectomy [3,4], though other research did not confirm this association [8]. Recently, downregulation of the *GCH1* gene by adeno-associated virus mediated expression of small hairpin RNA against *GCH1* was shown to reduce neuropathic pain hypersensitivity in rats [9]. Also, inhibition of GTP-CH1 was found to reduce cancer-induced pain in mice [10].

In contrast to the *GCH1* PP haplotype leading to moderate reduction of BH4 availability and only after stimulation, loss of function mutations in the *GCH1* gene causes reduced basal concentrations of BH4 and is associated with DOPA-responsive Dystonia (DRD). DRD is a rare movement disorder that manifest without hyperphenylalaninemia and the classical clinical characterisation are gait problems due to dystonia, mostly in the lower extremities with onset in childhood. The patients respond well to treatment with L-DOPA [11].

The hyperphenylalaninemia 1 (*hph-1*) mouse is a genetic model for DRD. The mutant mouse was originally generated by mutagenesis using the sperm mutagen N-ethyl-N´-nitrosourea [12], and is characterised by a marked decrease in baseline *GCH1* mRNA expression accompanied by a large decrease in both GTP-CH1 activity and BH4 synthesis. The *hph-1* mutation has been localised to an interval of 1.6-2.8 Mb on chromosome 14, containing the murine *GCH1* gene [13]. However, the exact location of the mutation is still undefined.

To the best of our knowledge it has not yet been reported whether mutations in the *GCH1* gene, leading to profound decrease in basal gene expression, affects pain sensitivity. Therefore, in the current study we investigated the *hph-1* mice in animal models of acute and inflammatory pain. We demonstrate that the mutant mice exhibited reduced inflammatory hypersensitivity, whereas acute responses to mechanical and heat stimuli were normal compared to wild type (WT) controls.

## Results

### Reduced BH4 concentrations in *hph-1* mice compared to WT mice

**Figure 1:**
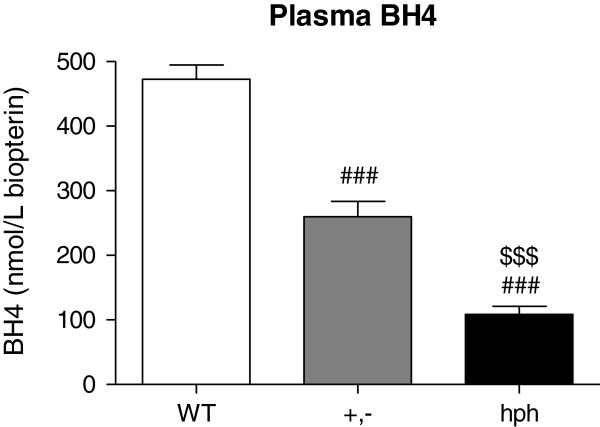
**Plasma concentrations of BH4 determined as biopterin (nmol/L) by reversed-phase HPLC.** The BH4 concentration was significantly reduced in both +,- and hph mice compared to WT mice (^###^*p* < 0.001) (n = 6 for WT and +,- mice, and n = 7 for hph mice). Moreover, hph mice displayed significantly lower BH4 concentrations compared to +,- mice (^$$$^*p* < 0.001). One-way ANOVA with pair-wise comparisons using the Fisher’s LSD test. Data are presented as mean + SEM.

Plasma BH4 concentrations were examined by high performance liquid chromatography (HPLC) analysis to evaluate if the *hph-1* mice used in this study had the expected phenotype of reduced BH4 synthesis [14]. The data showed that heterozygous *hph-1* (+,-) mice and homozygous *hph-1* (hph) mice had significantly lower BH4 concentrations compared to WT controls (^###^*p* < 0.001, Figure 1), with +,- mice having intermediate amounts of BH4. The plasma BH4 values were 472.8 ± 22.14 nmol/L, 259.6 ± 23.92 nmol/L and 108.5 ± 12.42 nmol/L for WT, +,- and hph mice, respectively. Hence, in plasma, heterozygous and homozygous animals have approximately 55% and 20% of normal BH4 concentrations, respectively.

### Lack of motor impairment and dystonia-like symptoms in *hph-1* mice

As the *hph-1* mouse is biochemically a model for DOPA-responsive dystonia (DRD) [15], the motor behaviour was examined in this strain. We found that the *hph-1* mice showed no signs of motor impairment nor any abnormal behaviour when carefully observing the animals. Also in the wire hanging test, both *hph-1* and WT mice were able to hang upside down for 120 sec, indicating normal neuromuscular function.

**Figure 2:**
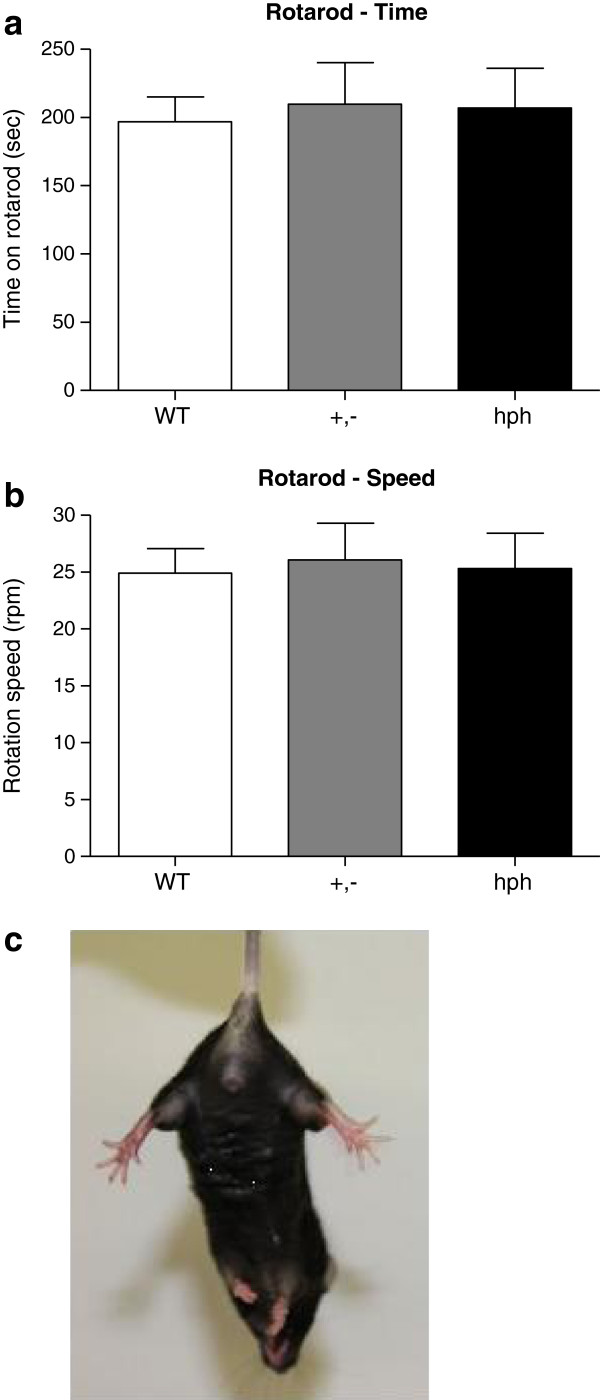
**No motor impairment nor dystonia-like symptoms were seen in +,- and hph mice.** (**a**) time walking on the rotarod (sec) and (**b**) rotation speed (rpm). n = 10, n = 8 and n = 6 for WT, +,- and hph mice, respectively. No difference in rotarod performance was found between genotypes (*p* = 0.92 and *p* = 0.95, respectively). One-way ANOVA with pair-wise comparisons using the Fisher’s LSD test. (**c**) hind-paw clasping; normal splaying of hind-paws was observed in hph mice as well as WT mice (not shown). Data are presented as mean + SEM.

In the rotarod test, +,- and hph mice had no difficulty maintaining balance on the rod compared to WT mice (Figure 2a-b). Both the time on rotarod (sec) and rotation speed (rpm) were not significantly different among genotypes (*p* = 0.92 and *p* = 0.95). The WT, +,- and hph mice stayed on the rotating rod for 197 ± 29 sec, 210 ± 31 sec and 207 ± 18 sec, respectively. In addition, dystonia-like symptoms were examined by suspending the animals by their tail for 15 sec, and this revealed that the mutant mice did not display any hind-paw clasping (Figure 2c). Overall, these observations demonstrate that *hph-1* mice are not associated with reduced motor function nor dystonia-like symptoms.

### The *hph-1* mutants exhibit normal pain behavioural responses to heat and mechanical stimuli

**Figure 3:**
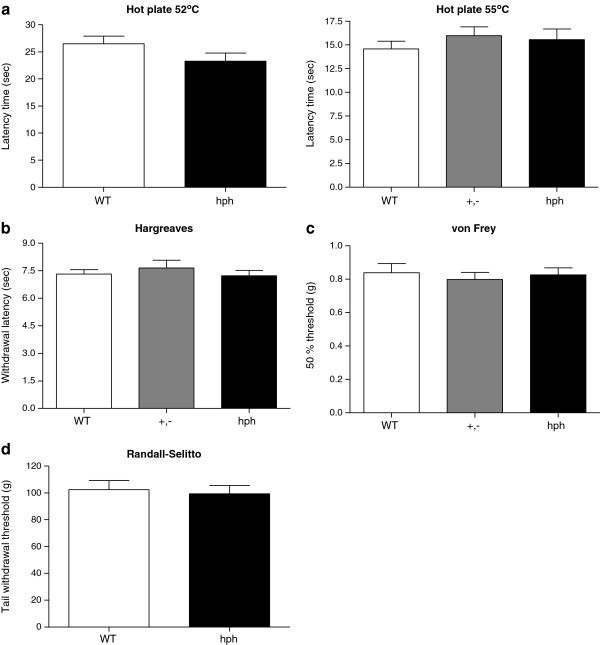
**Normal behavioural responses to heat and mechanical stimuli were observed in +,- and hph mice.** (**a**) Hot Plate; latency time to respond by hind-paw licking, lifting or flinching at a plate temperature of 52°C (n = 19) or 55°C (n = 12–17). No significant change was observed between genotypes (*p* = 0.13 and *p* = 0.54, respectively). (**b**) Hargreaves Test; withdrawal latencies to noxious heat stimuli (n = 7–15). No change in latency time was found among genotypes (*p* = 0.65). (**c,d**) von Frey and Randall Selitto; withdrawal threshold to mechanical stimulus (n = 10–16 and n = 13–15, respectively). *Hph-1* mice exhibited similar mechanical thresholds as WT mice (*p* = 0.92 and *p* = 0.68). Mann–Whitney *t*-test, two-tailed or one-way ANOVA with pair-wise comparisons using the Fisher’s LSD test. Data are presented as mean + SEM.

Acute heat sensitivity was assessed by two different assays; the hot plate test (Figure 3a) and the Hargreaves test (Figure 3b). In the hot plate test, the latency time to respond to noxious heat stimulation was not significantly different between *hph-1* mice and WT mice at neither 52°C (WT: 27 ± 1 sec and hph: 23 ± 2 sec, *p* = 0.13) nor 55°C (WT: 14.6 ± 0.8 sec, +,-: 15.9 ± 0.9 sec and hph: 15.5 ± 1.1 sec, *p* = 0.54). In the Hargreaves test, the paw withdrawal latencies of mutant mice to heat stimulation was also not different from WT responses (WT: 7.3 ± 0.2 sec, +,-: 7.6 ± 0.4 sec and hph: 7.0 ± 0.3 sec, *p* = 0.65).

Mechanical sensitivity was examined using the von Frey and Randall Selitto test (Figure 3c-d). In the von Frey test, the withdrawal threshold to mechanical stimulation was not significantly different between genotypes (WT: 0.84 ± 0.06 g, +,-: 0.79 ± 0.04 g and hph: 0.83 ± 0.04 g, *p* = 0.92). Similarly, the mean weight resulting in tail withdrawal in the Randall Selitto test was 102.4 ± 6.8 g for WT mice and 99.4 ± 6.2 g for hph mice, representing a non-significant difference between the groups (*p* = 0.68). Together, these behavioural observations suggest that reduction of BH4 synthesis in *hph-1* mice does not seem to change sensitivity to peripherally applied heat and mechanical stimuli in naïve mice.

### Reduced CFA-induced mechanical and heat hypersensitivity in *hph-1* mice compared to WT mice

In the complete Freund’s adjuvant (CFA) model, the baseline withdrawal thresholds to mechanical stimuli of naïve mice were not significantly different between genotypes (0.8 ± 0.07 g, 0.74 ± 0.03 g and 0.65 ± 0.06 g for WT, +,- and hph mice, respectively. *p* > 0.05). Moreover, the mean withdrawal latencies to heat stimuli were similar in hph mice and WT mice (9 ± 1 sec and 9 ± 1 sec, respectively).

**Figure 4:**
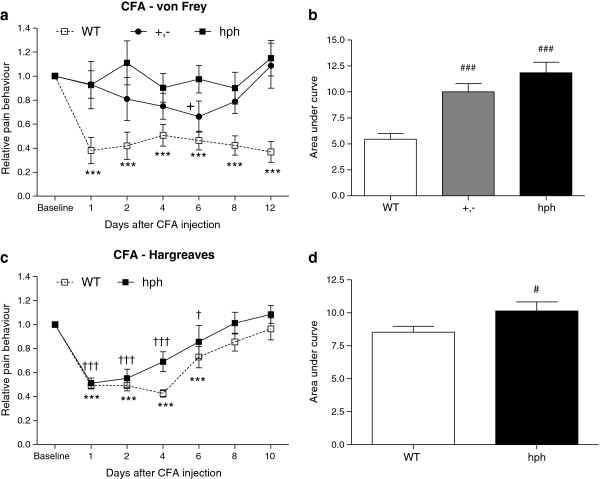
**Reduced hypersensitivity after CFA injection in +,- and hph mice.** (**a,c**) Time course of mechanical and heat hypersensitivity. CFA injection induced mechanical hypersensitivity in +,- and WT mice, but not in hph mice (n = 10–14). In the Hargreaves test, both WT mice and hph mice exhibited heat hypersensitivity after peripheral inflammation (n = 11–14). WT: ***p < 0.001, *+,-*: ^+^*p* < 0.05 and hph: ^†^*p* < 0.05 and ^†††^*p* < 0.001 versus baseline values (two-way RM-ANOVA with Fisher’s LSD test). (**b,d**) Area under curve analysis. A statistical significant difference in mechanical and heat hypersensitivity was found between *hph-1* mice and WT mice. ^###^*p* < 0.001, one-way ANOVA with pair-wise comparisons using the Fisher’s LSD test (log transformed data). ^#^*p* = 0.04, unpaired *t*-test, two-tailed (log transformed data). Data are presented as mean ± or + SEM.

Intraplantar injection of CFA into the hind-paw of WT mice induced a long-term mechanical hypersensitivity, starting on day 1 and persisting on day 12 (Figure 4a). The latter is indicated by reduced withdrawal thresholds to innocuous mechanical stimulation as compared to baseline values (***p < 0.001). In mutant mice, CFA injection evoked reduced withdrawal thresholds of the affected paw in +,- mice (^+^*p* < 0.05, Figure 4a), whereas almost no effect was observed in homozygous mice. Comparison of overall area under the curves (AUCs) revealed a significantly lower mechanical hypersensitivity of +,- and hph mice as compared to WT controls (^###^*p* < 0.001, Figure 4b). In addition, no statistical significant difference in pain-like responses was found between +,- and hph mice (*p* > 0.05). These results show that in homozygous mice, mechanical hypersensitivity was almost completely suppressed throughout the observation period compared to WT controls, whereas heterozygous mice displayed intermediate responses to CFA injection (Figure 4a). This suggests a genotype-dependent anti-hypersensitive effect, with the largest effect in the homozygous *hph-1* mice. Therefore, in subsequent experiments only WT and hph mice were tested.

Hind-paw injection of CFA also induced a significant reduction in paw withdrawal latencies in response to noxious heat stimuli in WT mice as compared to baseline values (***p < 0.001, Figure 4c). The heat hypersensitivity began at day 1 and lasted for 6 days after paw injection. In contrast to mechanical hypersensitivity, heat hypersensitivity was present in hph mice from 1 to 6 days after CFA injection (^†††^*p* < 0.001 and ^†^*p* < 0.05). However, a trend towards faster recovery and less heat hypersensitivity was seen in mutant mice compared to WT mice by day 4 (Figure 4c). Comparison of AUCs showed that hph mice exhibited significantly lower heat hypersensitivity than WT controls (^#^*p* = 0.04, Figure 4d).

To determine the degree of inflammation, the dorsal-plantar paw thickness was measured 24 hours after CFA inoculation (see Additional file 2). Injection of CFA increased the paw thickness in both genotypes as compared with baseline values (*p* < 0.001), and the data showed no difference in paw thickness between WT and hph mice (*p* > 0.05), indicating similar peripheral inflammation.

These data show that BH4 plays an anti-hypersensitive role in inflammatory pain in particular mechanical sensitivity following peripheral inflammation. For the heat hypersensitivity, the data suggest that BH4 may modulate pain sensitivity. However, this effect does not seem to be robust.

### Reduced acute nociceptive behaviour in response to formalin injection in hph mice

**Figure 5:**
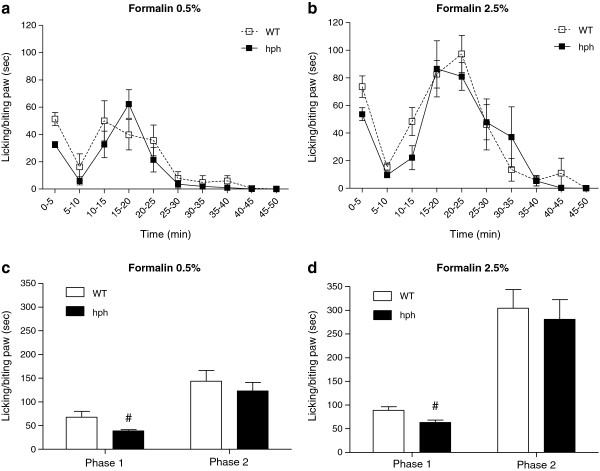
**Reduced formalin-induced pain behaviour in hph mice.** (**a**) and (**b**) time course after paw injection of 0.5 and 2.5% formalin. (**c**) and (**d**) total time licking and biting the paw during 0–10 min (phase 1) and 10–50 min (phase 2). A statistical significant reduction in spontaneous pain activity of hph mice was seen at both formalin-concentrations in the first phase compared to WT mice (0.5%: ^#^*p* = 0.014, n = 9 and 2.5%: ^#^*p* = 0.012, n = 9). Mann–Whitney *t*-test, two-tailed. No significant change in pain behaviour between genotypes was found in the second phase (0.5%: *p* = 0.48 and 2.5%: *p* = 0.69). Unpaired *t*-test, two-tailed. Data are presented as mean ± or + SEM.

Spontaneous nociceptive pain behaviours due to chemical stimuli were tested using the formalin test (Figure 5). Intraplantar injection of 0.5 and 2.5% formalin produced a biphasic response in both WT mice and hph mice, characterised by licking and biting the paw, with a first phase starting immediately after formalin injection and a second phase that began after 10 min and lasted for 50 min (Figure 5a-b).

Following injection of 0.5% formalin, a significant decrease in total time licking and biting the paw was seen in hph mice in the first phase compared to WT controls (hph: 39 ± 3 sec, WT: 68 ± 12 sec, ^#^*p* = 0.014, Figure 5c). On administration of 2.5% formalin a similar reduction in spontaneous pain behaviour was seen in the first phase (hph: 63 ± 5 sec, WT: 89 ± 7 sec, ^#^*p* = 0.012, Figure 5d). Regardless of the intensity of noxious chemical stimulus, no significant difference in spontaneous pain activity was found in the second phase between homozygous mutant mice and WT mice (0.5%: 123 ± 18 sec vs. 144 ± 22 sec, *p* = 0.48 and 2.5%: 281 ± 41 sec vs. 304 ± 39 sec, *p* = 0.69, Figure 5c-d).

These data demonstrate a significant effect of inherited BH4 reduction on formalin-evoked spontaneous pain responses. This was specifically observed in the first phase, suggesting that BH4 might modulate acute nociception induced by formalin injection.

### Reduced capsaicin-induced mechanical hypersensitivity but not spontaneous pain behaviour in hph mice compared to WT mice

**Figure 6:**
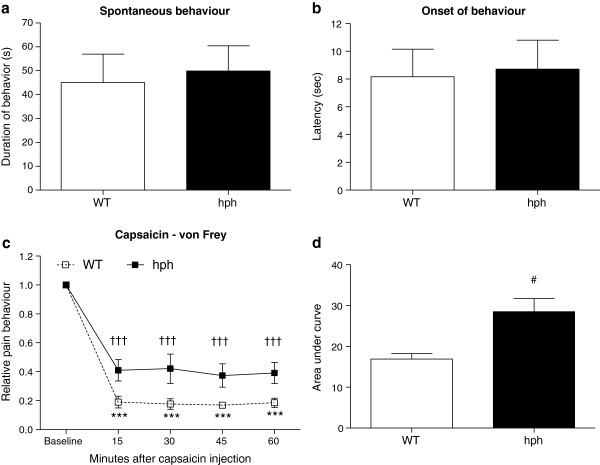
**Reduced capsaicin-induced mechanical hypersensitivity in hph mice, but normal spontaneous pain behaviours.** (**a**) Spontaneous pain responses; total time licking, biting and lifting the paw during 10 min after capsaicin injection (1 μg/paw). No difference was found between hph mice and WT mice (*p* = 0.46, n = 7–8). (**b**) Onset of pain behaviour. No differences in latency time was seen among genotypes (*p =* 1.00*,* n = 7–8). Mann–Whitney *t*-test, two-tailed. (**c**) von Frey time course; after capsaicin injection (1 μg/paw) both WT mice and hph mice developed mechanical hypersensitivity (n = 6–10). WT: ***p < 0.001 and hph: ^†††^*p* < 0.001 versus baseline values. Two-way RM-ANOVA with pair-wise comparisons using the Fisher’s LSD test. (**d**) Area under curve analysis. A statistical significant difference in mechanical hypersensitivity between hph mice and WT controls was found (^#^*p* = 0.04). Mann–Whitney *t*-test, two-tailed. Data are presented as mean ± or + SEM.

Intraplantar injections of capsaicin into the hind-paw evoked an immediate nocifensive behaviour characterised by licking, biting and lifting of the paw (Figure 6a). The duration of the spontaneous pain activity was not significantly different between hph mice and WT mice (45 ± 12 sec vs. 50 ± 11 sec, *p* = 0.46). In addition, the latency time to respond in hph mice was also similar to that observed in WT controls (8 ± 2 sec vs. 9 ± 2 sec, *p* = 1.00).

In the capsaicin test, the baseline withdrawal thresholds to mechanical stimulation of naïve mice were also similar between genotypes (*p* = 0.78). The mean withdrawal thresholds were 1.03 ± 0.09 g and 0.99 ± 0.04 g for WT mice and hph mice, respectively. Following capsaicin injection, mechanical sensitivity was evaluated within a 60 min observation period. Intraplantar injection of capsaicin (1 μg/paw) into the hind-paw induced a substantial mechanical hypersensitivity in both groups at all time points (WT: ***p < 0.001, hph: ^†††^*p* < 0.001, Figure 6c). When comparing the overall AUCs, a significant reduction in mechanical hypersensitivity was found in hph mice compare to WT mice (^#^*p* = 0.04, Figure 6d). At lower concentrations of capsaicin (0.25 and 0.5 μg/paw) both groups exhibited a clear mechanical hypersensitivity in the injected paw, however at these concentrations no difference in mechanical sensitivity was found between genotypes (*p* > 0.05, Table 1). Heat sensitivity following capsaicin injection was also examined, but in this case it was not possible to demonstrate presence of heat hypersensitivity in neither WT mice nor hph mice (see Additional file 3).

**Table 1:** Mechanical hypersensitivity following intraplantar injection of 0.25-1 μg/paw capsaicin in WT mice and hph mice

**Time of testing (min)**				
**Concentration**	**von Frey (g)**	**Baseline**	**30**	**60**
*0.25 μg/paw*				
WT mice	0.81±0.04	1.00±0.00	0.49±0.15^b^	0.39±0.07^b^
hph mice	0.78±0.04^a^	1.00±0.00	0.44±0.10^a,b^	0.47±0.07^a,b^
*0.5 μg/paw*				
WT mice	0.81±0.08	1.00±0.00	0.26±0.03^b^	0.28±0.11^b^
hph mice	0.80±0.10^a^	1.00±0.00	0.28±0.11^a,b^	0.26±0.03^a,b^
*1 μg/paw*				
WT mice	1.03 ± 0.09	1.00±0.00	0.18±0.04^b^	0.19±0.03^b^
hph mice	0.99 ± 0.04^a^	1.00±0.00	0.42±0.10^b,c^	0.39±0.07^b,c^

The results are expressed as relative pain behaviour (normalised to the baseline response of each mouse). For 0.25, 0.5 and 1 μg/paw n = 3–6, n = 4 and n = 6–10, respectively. The letter a, indicates non-significant differences when compared to WT mice (*p* > 0.05), the letter b, indicates significant differences compared to baseline, and the letter c, indicates significant differences relative to WT (*p* < 0.05). Unpaired *t*-test (two-tailed) or two-way RM-ANOVA with Fisher’s LSD test. Data is shown as mean ± SEM.

These data suggest that the effect of the mutation in hph mice on mechanical sensitivity is dependent on the intensity of the noxious stimulus induced by capsaicin. Moreover, the results revealed that reduced BH4 availability impaired capsaicin-evoked mechanical hypersensitivity, but not the immediate response induced by capsaicin itself.

## Discussion

In the current study, we used the *hph-1* mouse model deficient in GTP-CH1 to study the involvement of the *GCH1* gene in acute and inflammatory pain models. This included assays to determine mechanical and heat nociception as well as pain-like responses to intraplantar injection of chemical and inflammatory substances. We demonstrated that *hph-1* mice exhibited reduced mechanical and heat hypersensitivity following CFA, and reduced mechanical hypersensitivity after capsaicin injection compared to WT mice, whereas pain behavioural responses to heat and mechanical stimuli were normal in naïve mice.

### *GCH1* in mechano-and thermosensation

The present findings suggest that processes involved in the transmission of mechanical and heat stimuli in the non-sensitised organism are independent of GTP-CH1. This is consistent with previous studies in human carriers of the *GCH1* PP haplotype, and in rats following pharmacological inhibition with the GTP-CH1 inhibitor DAHP [4,5]. In rats, intraperitoneal administration of DAHP attenuated inflammatory pain behaviours, but it did not change mechanical and heat pain sensitivity in naïve animals [4]. Furthermore, differences in pain sensitivity between carriers and non-carriers of the *GCH1* PP haplotype were only observed after local skin inflammation or capsaicin sensitisation [3,5]. It was proposed that the reason for this observation is that differences in GTP-CH1 activity in carriers and non-carriers of the *GCH1* PP haplotype were only seen after stimulation with LPS [5]. In contrast to the human *GCH1* PP haplotype, the *hph-1* mice have significantly lower *GCH1* expression at baseline compared to WT mice. Baseline GTP-CH1 activity in homozygous *hph-1* mice is only about 10% of the WT mice [16] and in agreement with this a reduced BH4 concentration in blood was found in this study (Figure 1). Hence, our results indicate that even though baseline GTP-CH1 activity is reduced in *hph-1* mice it did not appear to influence mechanical and heat pain thresholds in naïve mice.

### *GCH1* in acute and inflammatory pain

After CFA injection, the homozygous mice did not develop mechanical hypersensitivity, while heat hypersensitivity was present in the mutant mice. This may suggest differential contribution of GTP-CH1 in the maintenance of mechanical and heat hypersensitivity. Several studies point to the involvement of different mechanisms of mechanical and heat hypersensitivity. Heat hypersensitivity is thought to be mediated by afferent C-fibers, whereas mechanical hypersensitivity involves activation of afferent Aβ-fibers [17]. Also studies using neuronal NOS knock-out mice reported profound loss of mechanical hypersensitivity, with no change in sensitivity to noxious heat stimulation [18]. Finally, the lack of correlation between mechanical and heat hypersensitivity in several pain models suggest that these are mediated by different neural mechanisms [19].

Following intraplantar formalin injection a significant reduction in spontaneous pain behaviour in hph mice was only seen during the first phase. Phase 1 is postulated to involve direct activation of nociceptors by formalin, probably mediated through chemical stimulation of the transient receptor potential cation channel subfamily A member 1 (TRPA1) [20,21], whereas phase 2 depends on inflammatory reactions in the peripheral tissue and/or central sensitisation of spinal cord neurons [22]. It has been proposed that the second phase results from the barrage of nociceptor inputs during phase 1 [23], hence in the mutant mice a reduction in pain behaviour during phase 2 was expected. In addition, our findings contrast with results from rats following pharmacological inhibition of GTP-CH1 activity, where attenuated pain behaviour was seen in both phases [4]. The reason for this discrepancy is not immediately apparent but it may relate to (i) difference in animal species and (ii) difference in the mechanisms following pharmacological inhibition versus genetic inhibition of GTP-CH1 activity. It is possible that although the nociceptive inputs from peripheral nerves are attenuated during phase 1 in the mutant mice, there is an increase or no change in excitability of spinal cord neurons in the second phase. Another potential explanation for the present observation is that sustained peripheral nerve activity rather than spinal sensitisation drives the nociceptive responses in phase 2. This is supported by previous studies showing that inhibition of pain responses during phase 1 with anaesthetics or opioids does not change the magnitude of nociceptive responses during phase 2 [24,25]. Nevertheless, the finding that spontaneous activity in *hph-1* mice was not influenced in the second phase after injection of both low and high formalin concentrations suggests that at least in *hph-1* mice, changes in GTP-CH1 activity is not critical for the processes driving the second phase of the formalin test.

Tegeder et al., 2006 showed that peripheral inflammation increased the BH4 concentrations in DRG compared to that of naïve rats, and that administration of DAHP reduced this excess BH4 production [4]. Reductions in BH4 synthesis in *hph-1* mice have been shown in several tissues, including brain, liver and lung [13,26,27], and GTP-CH1 activity is probably reduced in all tissues where it is normally expressed. Although the reduction of *GCH1* in *hph-1* mice have been shown to vary in different brain structures including dorsal raphe (51%), locus coeruleus (30%) and substantia nigra (39%), the *GCH1* expression was substantially reduced in all brain areas [28], consistent with decreased BH4 synthesis in the whole brain [26]. Furthermore, stimulation of cultured mast cells from *hph-1* mice with cytokines and administration of LPS increased the GTP-CH1 activity and BH4 synthesis, respectively. This indicates that enzyme activity can be induced in mutant mice, but absolute values were always markedly lower in *hph-1* mice than in WT mice [27,29]. Taken together, it is likely that the reduction of hypersensitivity following sensitisation is due to lower induction of *GCH1* and hence BH4 in DRG of *hph-1* mice compared to WT mice.

It is known that BH4 also contributes to the development of other types of pain conditions such as neuropathic pain and cancer-induced pain. Previous studies have demonstrated that systemic administration of DAHP has analgesic properties in three models of neuropathic pain and in two models of cancer-induced pain [4,10]. In humans, the *GCH1* PP haplotype has been associated with reduced risk of chronic pain after discectomy as well as a slow progression of pain in cancer patients [4,30]. The findings that inhibition of BH4 synthesis modulates pain sensitivity following inflammation, nerve injury and cancer imply a common mechanism of action of BH4 in these different pain states.

### Possible mechanisms of action

The mechanisms leading to pain and the sites of action of BH4 are still unknown. BH4 is not a neurotransmitter but a cofactor required for activity of a number of enzymes, including tyrosine and tryptophan hydroxylases and all isoforms of NOS. Accordingly, BH4 regulates the synthesis of serotonin, NO and catecholamines, all playing a complex role in nociceptive signalling. BH4 may therefore mediate noxious pain responses through mechanisms involving NO and/or biogenic amines [4,31]. Previous studies imply that BH4 acts partly through NO mediated mechanisms as injection of the GTP-CH1 inhibitor DAHP impaired excess NO production following nerve injury [4]. The implication of NO in development and maintenance of hypersensitivity in response to inflammation is well documented [18,32,33], and it has been shown that NO plays an essential role in both peripheral [33-36] and central [18] mechanisms of inflammatory pain. Reduced NOS activity and NO generation have previously been reported in *hph-1* mice determined in the brain as well as astrocytes and plasma [26,29,37]. Therefore, the alleviated mechanical hypersensitivity following CFA and capsaicin injection may possibly result from (i) decreased spinal neuronal NOS activity followed by decreased excess NO production and/or (ii) reduced NO-mediated peripheral sensitisation.

### Caveats of genetic mouse models

Although the use of genetic mouse models has their advantages, they are associated with important inherited caveats that need to be considered when interpreting the data [38]. As the *hph-1* mouse is a mutant model, having decreased GTP-CH1 activity during the development and throughout life, the role of BH4 in pain might be different from the ongoing role in the normal adult mouse. Therefore, genetic based studies might have different outcomes compared with pharmacological downregulation of GTP-CH1 in normal animals. However, it should be emphasised that this caveat also applies to humans carrying the *GCH1* PP haplotype.

The *GCH1* gene may also have multiple roles since it influences many biological systems. Consequently, disruption of this gene may have multiple effects unrelated to nociception such as change in motor function. The mutation in the *hph-1* mice share similar biochemical features as an autosomal dominantly inherited mutation in the human *GCH1* gene manifested as DRD [15]. DRD results from a reduction in BH4 biosynthesis and the prominent phenotype is gait problems due to dystonia [39]. Therefore, reduced BH4 in mice may also yield effects on motor behaviour, which can confound the pain behaviours in these animals. However, *hph-1* mice did not demonstrate any signs of motor impairment nor dystonia-like symptoms. Considering these observations, the differences in pain-related behaviours observed between *hph-1* mice and WT mice are not related to effects on motor function.

## Conclusions

In conclusion, our study supports previous findings that genetic functionally variants of the *GCH1* gene influence pain sensitivity after sensitisation. Furthermore, it revealed that even profound reduction in basal levels of *GCH1* activity did not affect nociceptive pain in naïve animals. The *hph-1* mice with profound decrease in GTP-CH1 activity displayed reduced pain-like responses after peripheral inflammation and sensitisation. As GTP-CH1 is the rate-limiting enzyme in the synthesis of BH4, these findings suggests that BH4 plays a role in modulating inflammatory pain that could be a therapeutic target to treat inflammatory pain conditions. Moreover, the *hph-1* mouse represents a new model to investigate the role of congenital *GCH1* deficiency in pain.

## Methods

### Animals

The *hph-1* mice have previously been generated and backcrossed for more than 20 generations into the C57BL/6JOlaHsd background (Harlan Laboratories, UK) [13]. Heterozygous male *hph-1* (+,-) mice from the Welcome Trust Center for Human Genetics were shipped to Taconic Denmark (Ejby, Denmark), where breeding took place. Initially, male heterozygous mice were mated with female C57BL/6JOlaHsd mice to produce heterozygous progeny, and these were then mated to generate WT, heterozygous and homozygous *hph-1* littermates. Later, animals were generated by homozygous breeding, no more than 4 generations. In this case control mice came from WT breeding pairs from the same colony as the *hph-1* mice. Genotyping was conducted as previously described [13]. Homozygous and heterozygous progeny were fertile in both sexes with a normal litter size. Moreover, a gross preliminary examination of general health revealed that *hph-1* mice mutants were healthy animals with a normal body weight compared to WT mice (Table 2).

**Table 2:** Average weight of WT, heterozygous and homozygous mice (gram)

**Genotype**	**Weight**	**SEM**
WT	24.49	0.51
+,-	24.00	0.33
hph	23.09	0.55

n = 29.

Experiments were performed on 7–12 weeks-old male mice, housed in colony cages in temperature-controlled environments, with unrestricted access to standard diet and tap water, and kept on a 12:12 h light–dark cycle. The behavioural tests were conducted during the daylight hours. Experiments were performed according to the ethical guidelines for the investigation of experimental pain with conscious animals [40], and were approved by the Danish Animal Experiments Inspectorate, Ministry of Food, Agriculture and Fisheries (No. 2009/561-1622). The experimenter was blinded to the animal genotypes during all experimental procedures.

### Determination of BH4 in plasma

BH4 was determined by isocratic HPLC analysis as the difference in biopterin (stable oxidation product of BH4) after acidic and basic oxidation with iodine [41]. Orbital blood was collected in tubes containing 10 μl of 180 mg/ml K_2_-EDTA (Sigma-aldrich) and 25 μl of 4% (w/v) dithiothreitol (Sigma-aldrich). Samples were then stored at room temperature for 3 h and protected from light before centrifugation at 2650 × g for 20 min [41]. The plasma samples were stored at – 80°C until analysis. Samples were then oxidised with a mixture of 1% (w/v) I_2_ (AppliChem, Kongens Lyngby, Denmark) and 2% (w/v) KI (Merck KGaA, Copenhagen, Denmark) in either 1 M HCl or 1 M NaOH for 1 h in the dark and at room temperature. Excess iodine was reduced by addition of 5% (w/v) ascorbic acid (Sigma-aldrich). The oxidised samples were centrifuged in Amicon Ultra Centrifugal filters (Ultracel –10 k; Millipore) at 5000 × g for 30 min. Biopterin content was determined in duplicate by reverse phase HPLC with fluorescence (Waters 474 Scanning Fluorescence Detector, Milford Massachusetts, USA) using a Nucleosil 100 C18 analytical column (5 μm, L × ID 250 × 4.6 mm; Varian) in conjunction with a pre-column (Nucleosil 100 C18 5 μm, ID 4.6 mm; Varian). The mobile phase consisted of 10 mM KH_2_PO_4_ in 5% (v/v) methanol (HPLC gradient grade; Sigma-aldrich) with pH adjusted to 4.5. The sample was injected in a volume of 20 μL and the analysis was run at a flow of 1 ml/min at ambient temperature. The peak area was used to quantify biopterin in comparison with external standards.

### Motor behavioural tests

The hanging wire test was performed using a wire cage lid, and the latency (sec) to fall off the wire lid was determined during 120 sec. Hind-paw clasping was tested to evaluate whether the mutation exhibited dystonic posture of the hind-paws. Each mouse was picked up by its tail and suspended for 15 sec to observe hind-paw clasping as previously described [42]. The rotarod test was used to test motor coordination and balance using an ENV-575 M Five Station RotaRod Treamill USB-Mouse (Med Associates inc., St. Albans, Vermont, USA). Mice were placed on an elevated rod (3.2 cm diameter) beginning its acceleration at 3.5 rpm and ending at 35 rpm over 5 min. Prior to testing, mice were trained for at least three times. Between trials animals were allowed to rest for 15 min. The time in sec on the rotarod and the rotation speed was recorded. Time periods where mice were passively rotating with the rod was subtracted.

### Hot plate test

Acute heat sensitivity was tested using a Hot Plate Analgesia Meter (Harvard Apparatus, Edenbridge, UK) as described by Eddy and Leimbach with few modifications [43]. On the day of testing, mice were allowed to habituate in their home cages for 60 min in the test room. After habituation, each mouse was placed one at a time in a transparent Plexiglas cylinder (13 cm high; diameter 19 cm) on a metal plate preheated to 52 or 55 ± 0.1°C, and observed until they responded by either hind-paw licking, lifting or flinching (whichever came first). Forepaw licking and lifting are common grooming responses and therefore not defined as nociceptive behaviour. The animals were removed immediately after showing a nociceptive response and were only tested once. A cut-off time of 40 sec was used to prevent tissue damage. The latencies to respond were taken for statistical analysis.

### Hargreaves test

Heat sensitivity to noxious stimuli was assessed with a plantar test device (Model 400 Heated Base from IITC Inc, Woodland Hills, Ca, USA) using the method of Hargreaves et al. [44]. Each mouse was placed in a square opaque Plexiglas chamber (12 cm high; 10 × 10 cm) on a glass surface (1.2 cm thick) at room temperature. The animals were first allowed to habituate for at least 60 min before measurements began. A mobile radiant heat source (located approximately 6 cm below the glass surface) was then aimed at the middle plantar surface of the hind-paw, and the heat stimulus was applied until the mouse made a clear nocifensive withdrawal of the paw. Testing during grooming or exploratory behaviour was avoided [45]. The intensity of the heat source was adjusted to 22% of maximal intensity ≈ 80.89 ± 0.6 mW/cm^2^. Each mouse was tested three times, and at least 1 min was allowed between consecutive measurements in the same paw. The heat sensitivity is expressed as the mean withdrawal latency time (sec). A cut-off latency time of 20 sec was used. Baseline measurements were obtained from each animal for at least 2 consecutive days prior to inflammation or sensitisation.

### von Frey test

Mechanical sensitivity was measured using von Frey monofilaments (ranging from 0.008 to 2.0 g, North Coast Medical Inc., Morgan Hill, Ca, USA) by the up-and-down method described by Chaplan et al. [46]. The mechanical sensitivity is recorded as 50% threshold (in grams), the force of the monofilament to which the animal responds in 50% of the stimuli. Each mouse was placed in an individual red Plexiglas cylinder (7.3 cm high; diameter 7.5 cm) on an elevated wire mesh floor (0.65 × 0.65 cm) allowing access to the plantar surface of the hind-paws. The animals were allowed a habituation period of at least 60 min. von Frey monofilaments were then applied perpendicularly to the middle plantar surface with sufficient force to cause filament bending and held for approximately 5 sec or until the hind-paw was withdrawn, defining a positive response. Lifting the hind-paw due to normal locomotory behaviour was ignored, and testing during deep sleep, grooming and exploring was avoided [45]. Baseline measurements to mechanical stimuli were performed in each animal for at least 2 consecutive days prior to inflammation or sensitisation.

### Randall Selitto test

Mechanical pain behaviour was measured using a Randall Selitto device (IITC Life Science Inc., Victory Blvd Woodland Hills, CA, USA). Briefly, mice were allowed to habituate in their home cages for 60 min in the test room. Each mouse was then placed one at a time in a transparent restrainer for at least 5 min before the application of increased pressure to the tail. Mice were tested three times on different locations starting from the middle of the tail to the root. At least 3 min was allowed between consecutive measurements. A cutoff of 300 g was used. The mechanical sensitivity is expressed as the mean withdrawal latency in grams.

### CFA-induced inflammatory pain

Persistent inflammatory pain was induced by injection of CFA (1 mg/ml *Mycobacterium tuberculosis*, Sigma-aldrich) into the hind-paw of mice. The mice were lightly restrained using a piece of cloth and 20 μl of CFA suspension was subcutaneously (s.c.) injected into the plantar surface of the right hind-paw using a GASTIGHT® 50 μl microsyringe (Hamilton Company, VWR, Denmark) with a 30^1/2^-gauge needle. Mechanical hypersensitivity after peripheral inflammation was then tested 1, 2, 4, 6, 8 and 12 days after injection, whereas heat hypersensitivity was tested 1, 2, 4, 6, 8 and 10 days after paw inflammation as described above. Paw thickness (mm) was measured after 24 hours using a digital caliper.

### Formalin test

Spontaneous nociceptive pain behaviours in response to chemical stimulation were measured using the formalin test described by Hunskaar et al. [47], with some modifications. Mice were placed within a glass cylinder (diameter 10.5 cm), and allowed to habituate for 60 min before behavioural testing began. Angled mirrors were placed behind and beside the glass cylinder to allow for an unimpeded view of the paws. After habituation, 20 μl of 0.5 or 2.5% (v/v) formalin solution (in isotonic saline) was injected into the right hind-paw as described above. The mice were immediately returned to the glass cylinder and a timer started to mark the beginning of the observation period. The total time spend licking/biting the right hind-paw during 60 min was measured with a stopwatch and recorded to the nearest second in 5 min intervals.

### Capsaicin test

Injection of capsaicin into the hind-paws of animals evokes spontaneous pain behaviour and hypersensitivity to mechanical and heat stimuli. Spontaneous nociceptive pain behaviours in response to capsaicin injection were measured as described by Sakurada et al. [48], with some modifications. The experimental procedure is similar to the formalin test described above. After habituation 10 μl of 1 μg/paw of capsaicin (Sigma-aldrich, Brøndby, Denmark) in 0.5% (v/v) ethanol and isotonic saline was injected s.c. into the plantar surface of the right hind-paw. The total time spend licking, biting and lifting the injected paw during 10 min was measured with a stopwatch and recorded to the nearest second.

Hypersensitivity to heat and mechanical stimuli was determined as described by Gilchrist et al. [49], with some modifications. Capsaicin 0.25, 0.5 or 1 μg/paw was injected s.c. into the plantar surface of the paw. Hypersensitivity was then tested at 15, 30, 45 and 60 min after capsaicin injection. Heat hypersensitivity was measured at the site of injection, whereas mechanical hypersensitivity was determined approximately 2 mm from the site of capsaicin injection.

In preliminary pilot studies (not shown), vehicle injections did not induce significant pain-like behaviours in the capsaicin test as well as the CFA and formalin pain models. Hence, in this study vehicle controls were not included.

### Data analysis

The concentrations of BH4 in plasma are expressed as nmol/L biopterin. Dihydrobiopterin (BH2), involved in the regeneration of BH4 (see Additional file 1), can also be oxidised to biopterin. During acidic oxidation both BH2 and BH4 are converted to biopterin, whereas basic oxidation involves the oxidation of BH2 only. Hence, the actual levels of BH4 are calculated as the difference in biopterin levels between acidic and basic oxidation.

The CFA and capsaicin data are expressed as relative pain behaviour. Each mouse was normalised to its own baseline value, i.e. expressed as index values with the baseline value defined as 1.0 for each mouse. Data were analysed using either unpaired *t*-test, one-way analysis of variance (ANOVA) or two-way repeated measured analysis of variance (RM-ANOVA) followed by pair-wise comparisons on the predicted means using the Fisher’s LSD test. Area under curve analysis was conducted using the trapezoidal rule. Statistical transformations were done where appropriate to meet the requirement of normal distribution and/or equal variances. The Mann–Whitney test was performed where appropriate. Data are presented as mean ± SEM or + SEM. *p* values less than 0.05 were considered significant. Statistical analysis was performed using SigmaPlot 11.0 (Systat Software Inc.) or GraphPad Prism 4.03 (GraphPad Software Inc.).

## Abbreviations

Hph-1: Hyperphenylalaninemia 1; BH4: Tetrahydrobiopterin; BH2: Dihydrobiopterin; GTP-CH1: Guanosine triphosphate cyclohydrolase 1; NOS: Nitric oxide synthase; NO: Nitric oxide; DRG: Dorsal root ganglion; DAHP: 2,4-diamino-6-hydroxypyrimidine; PP: “Pain-protective”; WT: Wild type; HPLC: High performance liquid chromatography; DRD: DOPA-responsive dystonia; CFA: Complete Freund’s adjuvant; TRPA1: Transient receptor potential cation channel subfamily A member 1; TRPV1: Transient receptor potential cation channel subfamily V member 1.

## Competing interests

The authors declare that they have no financial or non-financial competing interests.

## Authors’ contributions

AN designed and performed the experiments, analysed the data and wrote the manuscript. OJB and LBM coordinated the project, helped to interpret the data and edited the manuscript. LSD and ML carried out the motor behavioural studies. AMH, ATM, ED and TSJ revised the article. All authors have discussed, commented and approved the final manuscript.

## Supplementary Material

Additional file 1 ***De novo *pathway of BH4 biosynthesis and its functions.** BH4 synthesis proceeds from GTP via three steps catalysed by GTPCH1, PTS and SR. BH4 is an essential cofactor for the aromatic amino acid hydroxylases (PAH, TH and TPH) as well as for all isoforms of NOS. The regeneration pathway involves two steps catalysed by PCD and DHPR. The red line indicates the enzyme targeted in the *hph-1* mouse model. Abbreviations: GTPCH1, GTP cyclohydrolase 1; PTS, 6-pyruvoyltetrahydrobiopterin synthase; SR, sepiapterin reductase; PAH, phenylalanine hydroxylase; TH, tyrosine hydroxylase; TPH, trypthophan hydroxylase; NOS, nitric oxide synthase; AADC, aromatic L-amino acid decarboxylase; PCD, pterin-4-acarbinolamine dehydratase; DHPR, dihydropteridine reductase. Click here for file

Additional file 2 **Intact paw thickness in hph mice following CFA injection.** The dorsal-plantar paw thickness was measured using a digital caliper. A significant increase in paw thickness was found in both genotypes as compared with baseline values (n = 5). WT: ***p < 0.001 and hph: ^†††^*p* < 0.001. No significant difference was found between genotypes (*p* > 0.05). Two-way RM-ANOVA with pair-wise comparisons using the Fisher’s LSD test. Data are presented as mean + SEM. Click here for file

Additional file 3 **No heat hypersensitivity was observed following capsaicin injection in both WT and hph mice.** Heat sensitivity was examined before as well as 15, 30, 45 and 60 minutes after intraplantar injection of capsaicin. Capsaicin did not induce heat hypersensitivity in both WT and hph mice (*p* > 0.05, n = 4). Two-way RM-ANOVA with pair-wise comparisons using the Fisher’s LSD test. Data are presented as mean ± SEM. Click here for file
