# Supplementary figures and images for: Impaired behavioural pain responses in hph-1 mice with inherited deficiency in GTP cyclohydrolase 1 in models of inflammatory pain
Source: Mol Pain. 2013 Feb 19;9:5. doi: 10.1186/1744-8069-9-5 (PMC3626862; doi:10.1186/1744-8069-9-5)

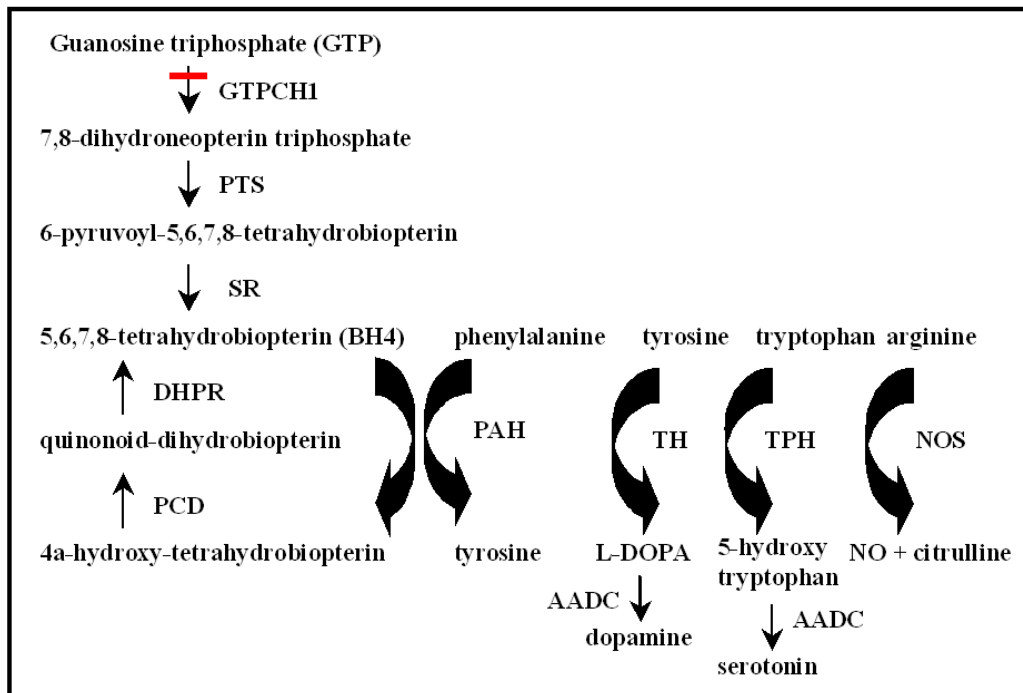

Additional file 1

Supplement: Additional file 1 — De novo pathway of BH4 biosynthesis and its functions. BH4 synthesis proceeds from GTP via three steps catalysed by GTPCH1, PTS and SR. BH4 is an essential cofactor for the aromatic amino acid hydroxylases (PAH, TH and TPH) as well as for all isoforms of NOS. The regeneration pathway involves two steps catalysed by PCD and DHPR. The red line indicates the enzyme targeted in the hph-1 mouse model. Abbreviations: GTPCH1, GTP cyclohydrolase 1; PTS, 6-pyruvoyltetrahydrobiopterin synthase; SR, sepiapterin reductase; PAH, phenylalanine hydroxylase; TH, tyrosine hydroxylase; TPH, trypthophan hydroxylase; NOS, nitric oxide synthase; AADC, aromatic L-amino acid decarboxylase; PCD, pterin-4-acarbinolamine dehydratase; DHPR, dihydropteridine reductase. [file 1744-8069-9-5-S1.pdf]

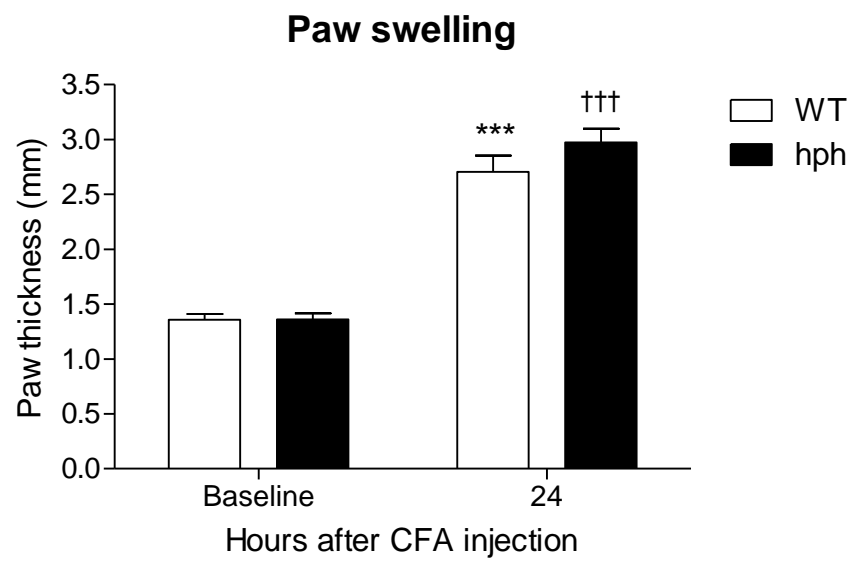

Additional file 2

Supplement: Additional file 2 — Intact paw thickness in hph mice following CFA injection. The dorsal-plantar paw thickness was measured using a digital caliper. A significant increase in paw thickness was found in both genotypes as compared with baseline values (n = 5). WT: ***p < 0.001 and hph: †††p < 0.001. No significant difference was found between genotypes (p > 0.05). Two-way RM-ANOVA with pair-wise comparisons using the Fisher’s LSD test. Data are presented as mean + SEM. [file 1744-8069-9-5-S2.pdf]

### Hargreaves

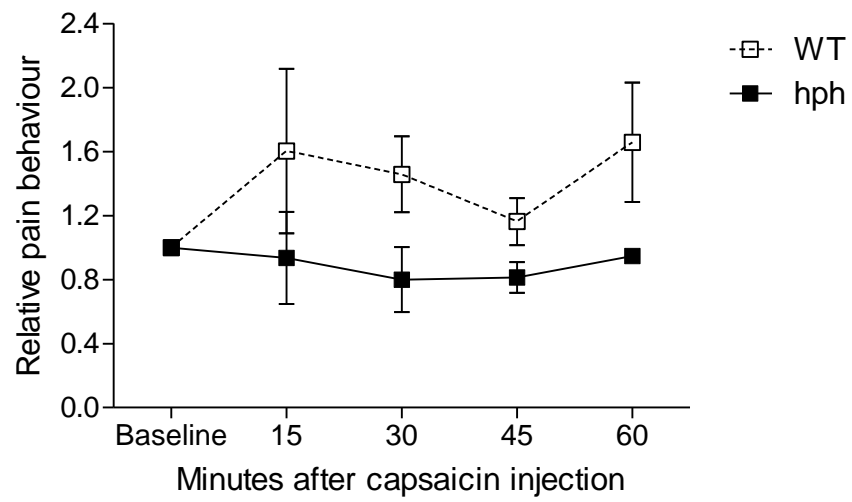

Additional file 3

Supplement: Additional file 3 — No heat hypersensitivity was observed following capsaicin injection in both WT and hph mice. Heat sensitivity was examined before as well as 15, 30, 45 and 60 minutes after intraplantar injection of capsaicin. Capsaicin did not induce heat hypersensitivity in both WT and hph mice (p > 0.05, n = 4). Two-way RM-ANOVA with pair-wise comparisons using the Fisher’s LSD test. Data are presented as mean ± SEM. [file 1744-8069-9-5-S3.pdf]
